# Supplementary material for: Development of a core outcome set for lower limb orthopaedic surgical interventions in ambulant children and young people with cerebral palsy: a study protocol
Source: BMJ Open. 2020 Mar 4;10(3):e034744. doi: 10.1136/bmjopen-2019-034744 (PMC7059521; doi:10.1136/bmjopen-2019-034744)
Supplement: Supplementary data [file bmjopen-2019-034744supp001.pdf]

Supplementary file 1: Search terms for each database

EMBASE (1974-Jan 2018)

1	cerebral palsy.mp. or exp *cerebral palsy/
2	("qualitative research*" or "qualitative study*" or "qualitative method*" or qualitative).mp.
3	(interview* or "focus group*").mp.
4	2 or 3
5	ambulatory.mp.
6	1 and 5
7	(parent* or caregiver* or "care giver*" or family* or carer*).mp.
8	1 and 7
9	(orthopedic surgery/ or pediatric surgery/ or exp *surgery/) mp.
10	4 and 8
11	1 and 4
12	11 and 9
13	(exp *"quality of life"/ or quality of life.mp.) and "cerebral palsy".mp.
14	4 and 13
15	(evaluat* or needs or concer* or satisfaction* or dissatisfaction* or perception* or experience* or expectation* or perceive* or perspective* or understanding or accept*).mp.
16	11 and 15

Medline (Ovid) (1946-Jan 2018)

1	cerebral palsy.mp. or exp *cerebral palsy/
2	("qualitative research*" or "qualitative study*" or "qualitative method*" or qualitative).mp.
3	(interview* or "focus group*").mp.
4	2 or 3
5	ambulatory.mp.
6	1 and 5
7	(parent* or caregiver* or "care giver*" or family* or carer*).mp.
8	1 and 7
9	(orthop?edic surgery/ or p?ediatric surgery/ or exp *surgery/).mp.
10	4 and 8
11	1 and 4
12	11 and 9
13	(exp *"quality of life"/ or quality of life.mp.) and "cerebral palsy".mp.
14	4 and 13
15	(evaluat* or needs or concer* or satisfaction* or dissatisfaction* or perception* or experience* or expectation* or perceive* or perspective* or understanding or accept*).mp.
16	11 and 15

PsycINFO (1806-Jan 2018)

1 cerebral palsy.mp. or exp *cerebral palsy/

2 ("qualitative research*" or "qualitative study*" or "qualitative method*" or qualitative).mp.

3 (interview* or "focus group*").mp.

4 2 or 3

5 ambulatory.mp.

6 1 and 5

7 (parent* or caregiver* or "care giver*" or family* or carer*).mp.

8 1 and 7

9 (orthop?edic surgery/ or p?ediatric surgery/ or exp *surgery/).mp.

10 4 and 8

11 1 and 4

12 11 and 9

13 (exp *"quality of life"/ or quality of life.mp.) and "cerebral palsy".mp.

14 4 and 13

15 (evaluat* or needs or concer* or satisfaction* or dissatisfaction* or perception* or experience* or expectation* or perceive* or perspective* or understanding or accept*).mp.

16 11 and 15

CINAHL (1806-Jan 2018)

1 (MM "Cerebral Palsy") OR "cerebral palsy"

2 (MH "Qualitative Studies") OR "qualitative research OR qualitative study OR qualitative methods OR qualitative"

3 (MM "Focus Groups") OR "interview AND "focus group*""

4 "ambulatory"

5 "parent* OR care giver* OR caregiver* OR family* OR carer*" AND (MM "Family")

6 2 and 3

7 1 and 4

8 (MH "Orthopedic Surgery") OR (MM "Pediatric Surgery") OR "orthopedic surgery OR paediatric surgery OR surgery"

9 1 and 5

10 6 and 9

11 1 and 6

12 6 and 8

13 (MM "Quality of Life") OR "quality of life"

14 11 and 13

15 "evaluate* OR needs OR concern* OR satisfaction* OR dissatisfaction* OR perception* OR experience* OR expectation* OR perspeive* OR perspective* OR understanding OR accept*"

16 11 and 15
